# Effect of Fingolimod on Lymphocyte Subsets in Patients With Relapsing Multiple Sclerosis

**DOI:** 10.7759/cureus.70715

**Published:** 2024-10-02

**Authors:** Cansu Sarıkaya, Gülçin Gacar, Hüsnü Efendi

**Affiliations:** 1 Department of Neurology, Maltepe University Faculty of Medicine, Istanbul, TUR; 2 Department of Stem Cells, Center for Stem Cell and Gene Therapies Research and Practice, Kocaeli University, Kocaeli, TUR; 3 Department of Neurology, Kocaeli University, Kocaeli, TUR

**Keywords:** b lymphocyte, fingolimod, lymphocyte subsets, multiple sclerosis, t lymphocyte

## Abstract

Background and aim

Multiple sclerosis (MS) is the most common chronic inflammatory demyelinating disease of the central nervous system (CNS). This study aims to evaluate the effect of fingolimod on T and B lymphocytes in relapsing-remitting multiple sclerosis (RRMS) patients.

Method

Multiple sclerosis patients were selected from patients who were scheduled to start medication at the outpatient clinic of Kocaeli University, Kocaeli, Turkey, between February 2019 and February 2022. Venous blood samples were obtained before starting medication from the patients who agreed to participate in the study and who were to start treatment, simultaneous clinical and neurologic examinations were performed and the Expanded Disability Status Scale (EDSS) was calculated. After the six-month treatment, venous blood samples were taken again. Lymphocyte subgroup analyses were performed in the flow cytometry laboratory.

Results

The study included 48 patients in the fingolimod group and 33 patients as controls. Flow cytometry analyses showed there was no significant difference between the two groups in the numbers and percentages of CD19+, CD20+, and CD22+ cells at baseline, while a significant decrease was observed in all of these parameters in the fingolimod group after the six-month treatment*.*

Discussion

Our findings support that the fingolimod treatment has significant effects on both lymphocyte counts and lymphocyte subgroup ratios. The results show the mechanism of action of fingolimod is unaccountable only through T lymphocytes, and it is effective in both B lymphocyte subgroups and T lymphocytes.

## Introduction

Multiple sclerosis (MS) is the most common chronic inflammatory demyelinating disease of the central nervous system (CNS) [1]. The etiology of MS is unknown clearly but is thought to be an autoimmune disease triggered by environmental factors and genetics [2,3].

Historically, MS has been classified as a T cell-mediated autoimmune disease. However, studies have revealed B cells make an important contribution. The contribution of B cells to the pathogenesis of MS is supported by the success of B cell-based immunotherapies [4].

The mechanism of action of fingolimod, which was approved for relapsing-remitting multiple sclerosis (RRMS), activates over sphingosine-1-phosphate receptors (S1PR) expressed on lymphocytes. Fingolimod is structurally similar to sphingosine, and a sphingosine-1-phosphate (S1P) analog is phosphorylated by sphingosine kinase 1/2 to become fingolimod-P. Similarly to S1P, fingolimod-P binds to the S1P1 receptor and renders the cell unresponsive to the exit signal. S1P receptor modulators cause the indirect antagonism of the function of the S1P receptor, as does fingolimod. It prevents autoreactive T lymphocytes from leaving secondary lymphoid organs, leading to a decrease in the number of lymphocytes reaching the CNS [5-7]. The studies on fingolimod have tried to explain the mechanism of action of fingolimod through T lymphocytes [8-11]. This study aims to evaluate the effect of fingolimod on T and B lymphocytes in RRMS patients.

## Materials and methods

Patients with RRMS were selected from patients who were scheduled to start medication at the MS outpatient clinic at Kocaeli University, Kocaeli, Turkey, between February 2019 and February 2022. The study was approved by the Kocaeli University clinical trial ethics committee on 19.02.2019 with file number 2019/70. Informed consent forms were obtained from all patients.

The inclusion criteria for the MS patients for the study were the following: diagnosis of clinically definite MS according to the 2017 McDonald criteria, age between 18 and 65 years of age, stable disease without a history of attacks in the last 30 days, and the ability to give informed consent. Exclusion criteria included primary or secondary progressive forms of MS, CNS disease in addition to MS, relapse within 30 days prior to including the study, severe infection within the last 30 days, and prior immunosuppressant therapy (azathioprine, cyclophosphamide, methotrexate, rituximab, mitoxantrone, mycophenolate mofetil, treatment within the last 30 days with corticosteroids, intravenous immunoglobulin, or plasma exchange).

51 patients were scheduled to commence fingolimod treatment, while 38 patients were scheduled to undergo RRMS follow-up. The control group was scheduled to initiate first-line therapies (teriflunomide, glatiramer acetate, and interferon-β 1a). Medication had not been initiated within the scope of the study. We determined the washout period for the patients’ former medications. Venous blood samples were obtained before starting medication from the patients who agreed to participate in the study and who were to start treatment, and simultaneous clinical and neurologic examinations were performed and the Expanded Disability Status Scale (EDSS) was calculated. After the six-month treatment, venous blood samples were taken again, and EDSS and attacks were recorded. The flow cytometry method was applied for lymphocyte subgroup determination in venous blood samples.

Flow cytometry

For whole blood staining, 2 ml of blood was taken into an ethylenediamine tetraacetic acid (EDTA) tube that was divided into 5 ml tubes (BD Falcon, Becton, Dickinson, and Company, Franklin Lakes, NJ) as 100 microliters. They were later vortexed after adding specific fluorescein isothiocyanate (FITC), phycoerythrin (PE), monoclonal antibodies conjugated with APC, PerCp 5.5; CD3 (T cell receptor, FITC), CD4 (T-helper cell receptor, PerCp 5.5), CD8 (cytotoxic T cell receptor, FITC), CD5 (T1, ly-1 signaling molecule, PerCp 5.5), CD10 (N-cadherin, common lymphocytic leukemia antigen, CALLA, PE), CD19 (co-stimulatory receptor on mature B cell, APC), CD20 (type III transmembrane protein, pre-B phase, PE), CD22 (SIGLEC family of lectins, mature B cells) protein, PerCp 5.5), CD16 (natural killer (NK) cell protein, FcγRIII), CD56 (neural cell-adhesion molecule, NCAM, NK cells marker, FITC), CD45 (protein tyrosine phosphatase, receptor type C, PTPRC, leukocyte common antigen, cells marker of hematopoietic origin, APC), and 2.10-20 microliters of appropriate isotype controls. The samples were incubated for 20 minutes at room temperature in the dark. Next, we added 2 milliliters of lysis buffer (BD FACS Lysing Solution 10x Concentrate Catalog No. 349202 (Becton, Dickinson, and Company) attained 1x concentration by distilled water) in order to burst the erythrocytes. The samples were revortexed and incubated for eight minutes at room temperature. Then, they were centrifuged at 1800 rpm for five minutes. After centrifugation, the supernatant was poured out. After adding cell wash solution (phosphate-buffered saline (PBS) containing 0.1% sodium azide), they were revortexed and centrifuged at 1800 rpm for five minutes. After removing the supernatant, the samples were resuspended with 500 microliters of cell wash. Cell suspensions were read on a BD FACSCalibur (Becton, Dickinson, and Company) flow cytometer.

Statistical analysis

Statistical analysis was performed using the IBM SPSS Statistics software, version 20.0 (IBM Corp., Armonk, NY). Normal distribution was evaluated using the Shapiro-Wilk test. We used an independent sample t-test and a Mann-Whitney U test for in-group comparisons. Time-dependent changes in variables were analyzed using the Wilcoxon signed-rank test and paired t-test. Relationships between categorical variables were evaluated by chi-square analysis. In testing of two-way hypotheses, p<0.05 was considered sufficient for statistical significance.

## Results

Demographic data

The study included a total of 89 patients followed up at the MS outpatient clinic at Kocaeli University. Two groups were formed of patients who planned to start fingolimod and patients who would receive non-fingolimod treatment. At the end of the study, five patients who were unable to come to the control group due to the COVID-19 pandemic were excluded from the study. The fingolimod group was finalized with 48 patients and the non-fingolimod control group with 33 patients. Of the control group, 18 (54.5%) were to be treated with teriflunomide, 8 (24.2%) with glatiramer acetate, and seven (21.1%) with interferon-β 1a.

There was no significant difference between the two groups in terms of gender and age (p = 0.685 for gender, p = 0.108 for age). The baseline EDSS of all patients ranged from 0 to six, with a median value of 1.5 (1.00-2.00) in the fingolimod group and 1.0 (1.00-2.00) in the control group. There was no significant difference between the two groups in terms of baseline EDSS scores (p = 0.116). The six-month EDSS averages showed no significant increase in disability was observed in both groups (p = 0.317 for the fingolimod group, p = 0.705 for the control group). Sociodemographic data are presented in Table 1. 

**Table 1:** Sociodemographic data ^a^Mean ± standard deviation, ^b^Median (25^th^-75^th^ percentile); *Independent sample t-test, **Chi-square test, ***Mann-Whitney U test EDSS: Expanded Disability Status Scale

Variables	Fingolimod group (n=48)	Control group (n=33)	p-value
Age^a^	35.92±9.98	32.39±8.93	0.108*
Gender, n (%) Female	38 (79.2)	24 (72.7)	0.685**
Gender, n (%) Male	10 (20.8)	9 (27.3)	0.685**
Baseline EDSS^b^	1.5 (1.00-2.00)	1.0 (1.00-2.00)	0.116***
Sixth-month EDSS^b^	1.5 (1.00-2.00)	1.0 (1.00-2.00)	0.215***

Immunological data

After the flow cytometry examinations, no significant differences were observed between the lymphocyte counts and percentages of the fingolimod and control groups before the treatment. However, we observed a significant decrease in lymphocyte counts and percentages of the two groups measured after six months. The decrease was more pronounced in the fingolimod group (p<0.001). For the number of CD5+ cells in some subgroups of T lymphocytes and B lymphocytes, there was no significant difference between the two groups before the treatment. However, there was a significant decrease in the number of CD5+ cells in the fingolimod group compared to the control group in the values measured after the sixth month of the treatment. In the analysis of CD3, a T lymphocyte surface marker, we found similar numbers and percentages for the two groups at baseline. In the values measured after the sixth month of treatment, we observed the number of CD3+ cells decreased significantly in the fingolimod group (p<0.001).

There were no significant differences between the two groups in the pre-treatment myeloid and monocyte percentages. However, we observed an increase in the post-treatment myeloid and monocyte percentages in the fingolimod group (p<0.001 for myeloid, p=0.010 for monocytes).

In the first analyses, we found similar rates for the number and percentage of CD45+ cells showing all nucleated cells. In the evaluations following the sixth month of treatment, we observed the number of CD45+ cells decreased statistically significantly in the two groups. The CD45 percentage decreased significantly in the fingolimod group only. Comparing the two groups, we observed the decrease in the fingolimod group was greater than that in the control group (p<0.001).

**Figure 1:**
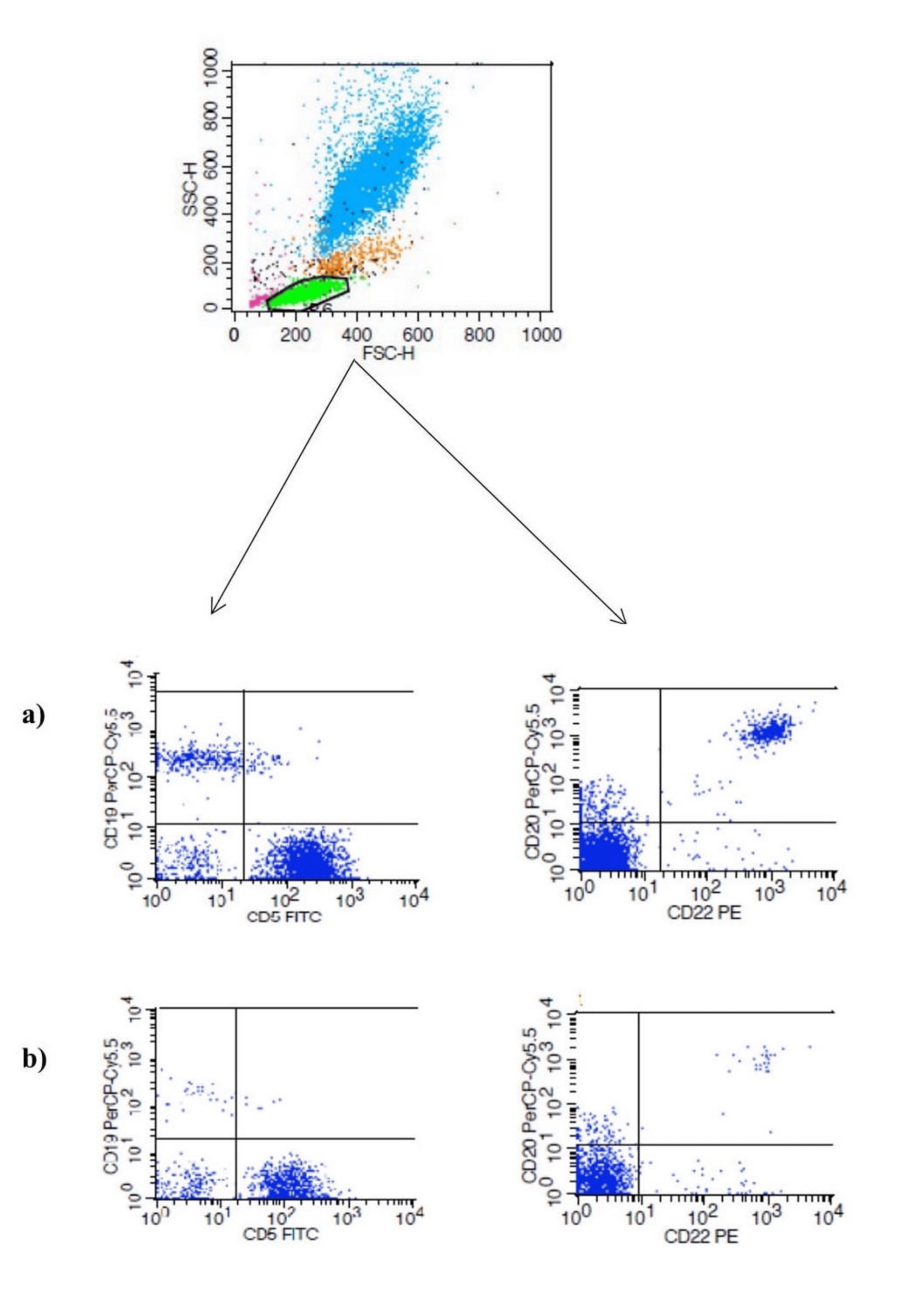
Gating process of lymphocyte and B cell subtypes in flow cytometry: (a) distribution of pre-treatment CD5+, CD19+, CD20+, and CD22+ cell subtypes; (b) redistribution of cell subtypes after six months of fingolimod treatment

**Figure 2:**
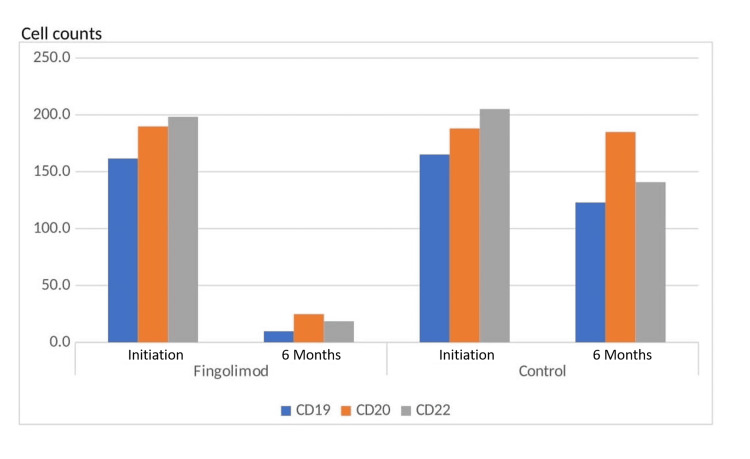
CD19+, CD20+, and CD22+ cell counts at the baseline and six months for both fingolimod and control groups

In the analysis of B lymphocyte surface markers CD19, CD20, and CD22, there were no significant differences between the two groups in numbers and percentages at the beginning. Following the sixth month of treatment, the analyses showed there was a significant decrease in CD19+, CD20+, and CD22+ cell counts and percentages in the fingolimod group compared to the control group (p< 0.001). Figure 1 and Figure 2 show the data before treatment and six months after treatment in CD 19+, CD 20+, and CD 22+ cell counts.

There was no difference between the two groups in the initial analysis of CD10+ cells expressed mainly in immature B lymphocytes. The decrease in the CD10 count in the fingolimod group was remarkable in the post-treatment evaluations (p<0.001). The evaluation of the number of CD5+ cells in T lymphocytes and some subgroups of B lymphocytes showed there were no significant differences between the two groups before the treatment. Following six months of treatment, there was a significant decrease in the number of CD5+ cells in the fingolimod group compared to the control group (p<0.001).

**Figure 3:**
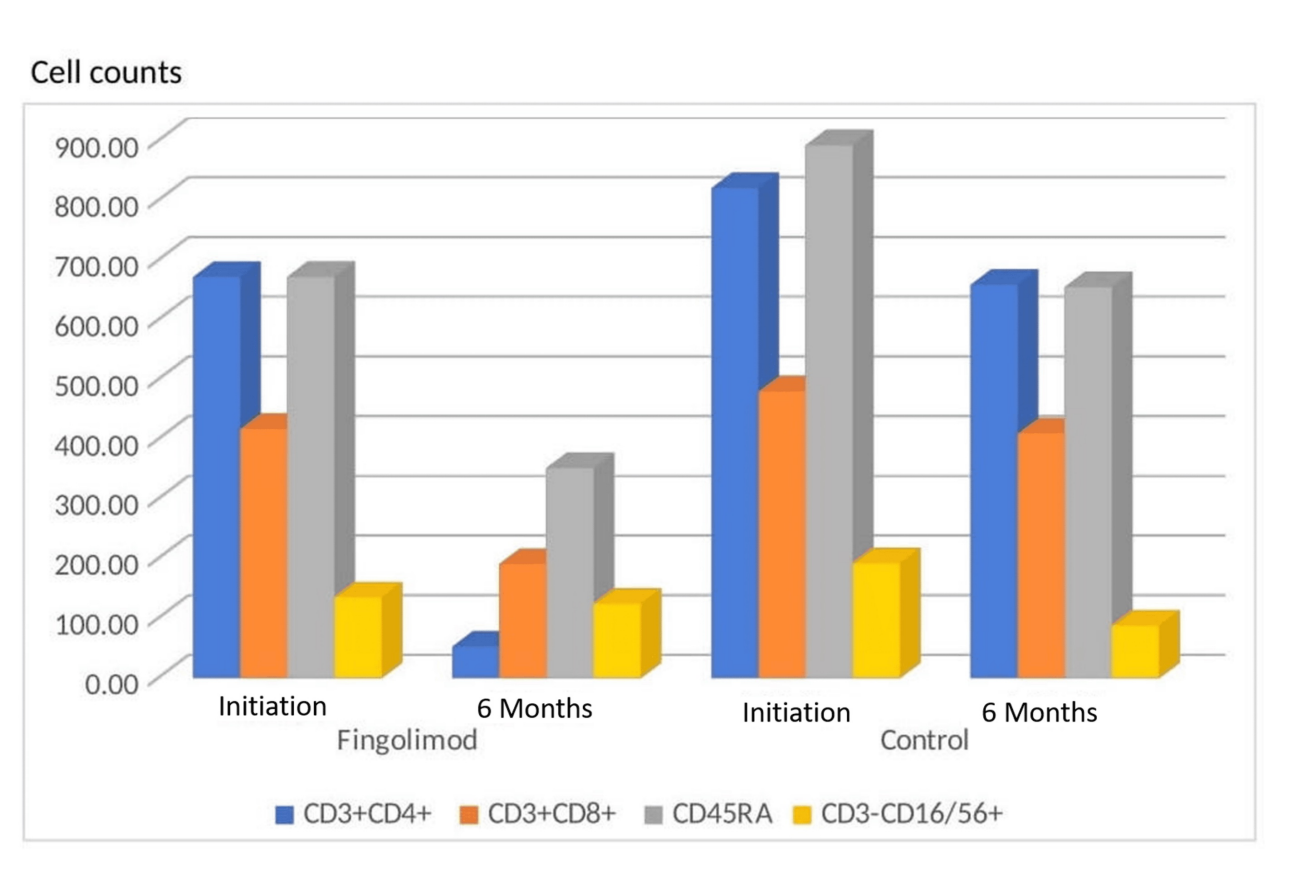
CD3+CD4+, CD3+CD8+, CD45RA, and CD3-CD16/56+ NK cells count at baseline and six months for both the fingolimod and controls groups. NK: natural killer

In the analysis of CD3, a T lymphocyte surface marker, the numbers and percentages were similar in the fingolimod and control groups at baseline. The values measured after the six months of treatment showed the number of CD3+ cells decreased significantly in the fingolimod group (p<0.001). The numbers and percentages of CD3+ CD4+ T helper cells and CD3+ CD8+ T cytotoxic cells were observed at similar rates in fingolimod and control groups before treatment. It was observed that the number of CD3+ CD4+ and CD3+ CD8+ cells decreased in both groups after treatment (Figure 3). The decrease in the fingolimod group differed significantly compared to the control group (p<0.001). 

When the CD4/8 ratio was examined, it was observed that this ratio deteriorated in favor of CD8 after the sixth month in both groups, and this effect was much higher in the fingolimod group. The median values for the fingolimod group before and after treatment were 1.45 (1.12-2.17) and 0.20 (0.20-0.40); the median values for the control group were 1.40 (1.10-1.80) and 1.60 (1.20-1.90) (p< 0.001).

In the analyses of CD45RA, a naive T cell marker, there was no significant difference between the two groups at the first visit, while a significant decrease was observed in the number and percentage of CD45RA cells in the fingolimod group after the six months (p<0.001).

CD3-CD16/56+ NK cells were found to be similar in number and percentage at the beginning. A statistically significant increase was found in the percentage of CD3-CD16/56+ NK cells in the fingolimod group in the post-treatment analysis (p<0.001). It was observed that there was a decrease in the number of cells. However, this decrease did not reach the level of statistical significance (p = 0.126).

## Discussion

Fingolimod, the first oral therapeutic molecule approved for RRMS, acts as an antagonist of S1PR, which is required for the exit of T lymphocytes from lymph nodes.

Lymphopenia is an integral part of fingolimod therapy based on its unique mechanism of action. The results of early experiments with fingolimod reported approximately 70% reduction in lymphocyte count due to entrapped lymphocytes in lymphoid tissue, and wide interindividual differences were discussed [12-14]. In our study, a significant decrease was observed in the total lymphocyte count, similar to the literature. The increase in myeloid and monocyte percentages in the patients receiving the fingolimod treatment was attributed to the decrease in the number and percentage of lymphocytes.

The cytometry results indicated a significant decrease in the number of CD45+ cells, which represented all nucleated cells, in the patients using fingolimod. Examining the lymphocyte subgroups of the patients, in CD3+ cell analyses representing T lymphocytes, we found a significant decrease in the number and percentage of CD3+ and CD3+ CD4+ helper T cells in the fingolimod group. Although not as strong as CD4+ T-cell numbers, CD3+ CD8+ T-cell counts also decreased in MS patients treated with fingolimod.

Although the dominant role of CD4+ T cells in MS has long been emphasized, more recent works indicate that CD8+ T cells B cells also play a critical role in disease development and actually comprise a proportion of the CNS infiltrating cells. CD8+ cells are reported to be predominant in the CNS lesions of MS, although compositions of cellular infiltrates vary greatly, depending on the types and stages of this disease.

Recent data have suggested that CD8+ T cells are involved in a second stage of CNS damage during relapses and in the chronic phase. The high number of CD8 T cells in the perivascular sheath and parenchymal lesions in postmortem studies provides evidence for the role of these cells in pathogenesis. In light of these results, we suggest the change in CD8+ cells contributes to the efficacy of fingolimod.

The examination of percentages shows CD8+ cells increased in percentage, and thus the CD4/CD8 ratio deteriorated in favor of CD8. This result infers the fingolimod treatment was also effective on CD8+ cells, but CD8+ cells were not trapped in lymphoid tissue as much as CD4+ cells; that is why there was a relative increase in percentage. As another result, we observed fingolimod treatment affected CD4 + T lymphocytes more than CD8 + cells, which is attributable to CD4+ T lymphocytes containing more CCR7 positivity. CCR7 is a chemokine involved in T cell recruitment in lymph nodes [15,16].

In our study, we also detected a decrease in the number of CD45RA+ T cells and found fingolimod was effective on naive T cells. Similar to our study, it has been shown in RRMS patients that a significant decrease was observed in CD3+, CD4+, and CD8+ cell counts as of the third month following the fingolimod treatment [8] [17]. A relative increase was observed in the number of granulocytes and monocytes with no significant effects.

Based on immunopathogenesis, in the subgroup analyses for investigating the effects of fingolimod on B lymphocytes, similar numbers were observed for CD19+, CD20+, and CD22+ cells in the fingolimod and control groups before the treatment. However, in the analyses performed six months after the start of treatment, a significant decrease in cell numbers and percentages was observed in the fingolimod group compared to the control group. A significant decrease was also observed in the number of CD10+ cells showing immature B lymphocytes and CD5+ cells in some B lymphocyte subgroups. Fingolimod both reduces the number of autoreactive lymphocytes in the periphery and changes the composition of B cell subsets. Another study included 40 RRMS patients who were grouped into two groups according to the use of natalizumab. The percentages of CD3+, CD4+, CD20+, CD19+, memory T cells, naive T cells, memory B cells, regulatory B cells, NK cells, and cytokine-producing cells (IFN, IL-17, and IL-2) decreased significantly after the treatment. After six months of fingolimod treatment, helper T cells were positively correlated with memory and regulatory B cells. A negative correlation was found between naive B cells and NK cells. Cytotoxic T cells were positively correlated with NKT cells and negatively correlated with NK cells [18]. No patients using such drugs, which may have a significant effect on lymphocyte subgroups, as natalizumab, ocrelizumab, rituximab, and alemtuzumab before transitioning to the fingolimod treatment, were included in our study. We found a significant decrease in CD3+, CD4+, CD19+, and CD20+ cell counts and percentages. Unlike this study, a decrease was observed in CD45+, CD45RA+, CD22+, CD10+, and CD5+ B lymphocyte counts. We observed a significant decrease in the percentage of CD5+ cells and no significant change in the percentage of CD10+ cells.

Natural killer cells are today known to play a role in MS immunology. The dysfunction of NK cells is probably associated with disease activity [19]. In our results, we detected a decrease in absolute NK cell count with fingolimod treatment, but it was not at a statistically significant level. There was a statistically significant decrease in the percentages.

Natural killer cell recirculation is mediated by S1PR1 and S1PR5. S1PR5 is allegedly less sensitive to fingolimod than S1PR1 used by other lymphocyte subtypes [20] [21]. This fact explains why NK cells are less affected than T and B lymphocytes.

Study limitations

Since our study had a small number of patients, we think that studies with a larger number of patients will contribute to this issue.

## Conclusions

Our findings support that fingolimod treatment has significant effects on both lymphocyte counts and lymphocyte subgroup ratios. The results show the mechanism of action of fingolimod is unaccountable only through T lymphocytes, and it is effective in both B lymphocyte subgroups and T lymphocytes.

Considering immunopathogenesis, we assert that the specific changes made by fingolimod in the distribution of lymphocyte subsets contribute to the treatment efficacy of fingolimod. The effect of fingolimod on B lymphocytes is promising in terms of B cell-mediated diseases.
